# Early Detection of Chronic Kidney Disease in Men Using Lifestyle and Demographic Indicators: A Machine Learning Approach for Primary Healthcare Settings

**DOI:** 10.3390/healthcare14030405

**Published:** 2026-02-05

**Authors:** Mc Neil Valencia, Jun Kim, Zeeshan Abbas, Seung Won Lee

**Affiliations:** 1 Department of Precision Medicine, School of Medicine, Sungkyunkwan University, Suwon 16419, Republic of Korea; hy.neilll@skku.edu; 2 Department of Metabiohealth, Institute for Cross-Disciplinary Studies, Sungkyunkwan University, Suwon 16419, Republic of Korea; ttr3515@skku.edu; 3 Department of Artificial Intelligence, Sungkyunkwan University, Suwon 16419, Republic of Korea; 4 Personalized Cancer Immunotherapy Research Center, School of Medicine, Sungkyunkwan University, Suwon 16419, Republic of Korea; 5 Department of Family Medicine, Kangbuk Samsung Hospital, School of Medicine, Sungkyunkwan University, 29 Saemunan-ro, Jongno-gu, Seoul 03181, Republic of Korea

**Keywords:** chronic kidney disease, machine learning, AdaBoost, lifestyle factors, demographic factors, dietary, serum creatinine, albuminuria, eGFR, ACR

## Abstract

**Background/Objective:** Chronic kidney disease (CKD) is a major global health concern associated with significant morbidity, mortality, and healthcare burden. This study aimed to develop an explainable machine learning framework that integrates lifestyle, sociodemographic, and biochemical factors for early CKD risk prediction among middle-aged men using public health survey data. **Methods**: Data from 968 male participants were preprocessed by removing missing values, deriving eGFR and ACR, and labeling CKD status. Five machine learning algorithms, (i.e., Random Forest, AdaBoost, Naïve Bayes, SVM, and XGBoost) were trained and evaluated using accuracy, precision, recall, and F1-score. Model interpretability was assessed using SHAP, LIME, Boruta, and Pearson’s correlation analyses. **Results**: AdaBoost yielded the best performance (accuracy = 0.7258, F1 = 0.6457, recall = 0.6923), with robust generalization confirmed by the precision–recall curve (AP = 0.715). SHAP and LIME revealed that serum creatinine, blood urea nitrogen, urinary creatinine, and age were major predictors, whereas lifestyle and metabolic indicators such as BMI, sodium and sugar intake, and sleep duration emerged as secondary factors for CKD. **Conclusions**: This study demonstrates the effectiveness of an explainable machine learning model that integrates lifestyle, sociodemographic and biochemical data for early CKD prediction among middle-aged men. The AdaBoost-based framework shows strong potential for implementation as a clinical decision-support tool within EHR systems and may contribute to personalized and preventive interventions. It emphasizes the growing importance of modifiable behaviors in kidney disease development and supports future work involving multiple cohorts and temporal model expansion to improve risk stratification for individuals at risk of kidney disease.

## 1. Introduction

Chronic kidney disease (CKD) is a major global public health concern characterized by a progressive and irreversible decline in kidney function. As a chronic condition with multifactorial causes and long-term consequences, CKD requires systematic classification and staging to guide risk assessment, clinical decision-making, and disease management [1]. Accordingly, CKD is classified based on its underlying cause, glomerular filtration rate (GFR) category (G1–G5), and albuminuria category (A1–A3). This classification framework plays a critical role in evaluating disease severity and predicting clinical outcomes by focusing on two key dimensions: the level of kidney filtration function and the extent of kidney damage reflected by albuminuria.

Specifically, GFR categories range from G1 (normal or high, ≥90 mL/min/1.73 m^2^) to G2 (60–89 mL/min/1.73 m^2^), which indicates mildly decreased function relative to young adult levels, to G5 (kidney failure, <15 mL/min/1.73 m^2^). Albuminuria categories range from A1 (normal to mildly increased, Albumin Excretion Rate (AER) < 30 mg/24 h) to A3 (severely increased, AER > 300 mg/24 h) [2]. Clinically, CKD is defined by various markers of kidney damage, such as reduced GFR and an albumin-to-creatinine ratio (ACR) greater than 30 mg/g persisting for at least three months or more (Table 1 and Table 2). As kidney function progressively declines, patients may ultimately require renal replacement therapy, including dialysis or kidney transplantation, underscoring the serious and often life-altering nature of the disease.

Beyond its clinical definition, CKD poses a substantial burden on global health systems due to its high morbidity and mortality rates, particularly among individuals with diabetes and hypertension [3,4]. Global Burden of Disease (GBD)-based analyses estimate that CKD affected approximately 670–675 million people worldwide, with global prevalence around 8000 cases per 100,000 population (about 8%), ranking approximately as the 9th–12th leading cause of death globally in 2021. Roughly 1.5 million were deaths attributed to CKD in 2021 [5], with projections indicating that global CKD deaths will exceed 1.8 million annually by 2030 if current patterns persist. Notably, nearly 85% of affected individuals live in low- to middle-income countries [6]. The growing prevalence and worsening outcomes of CKD can be attributed to a complex interplay of non-modifiable and modifiable risk factors. Non-modifiable factors include age, sex, and race, while modifiable factors encompass hypertension, diabetes, obesity, genetic predisposition, proteinuria, albuminuria, dyslipidemia, and environmental influences such as high dietary salt intake [7,8]. Understanding the correlations of these risk factors is essential for identifying high-risk populations and designing targeted prevention strategies.

Among these factors, sex-related differences in CKD prevalence and progression have drawn increasing attention. Epidemiological studies indicate that CKD is more prevalent in women than in men in most countries. For example, a population-based analysis by Carrero et al. [9] across 21 countries demonstrated consistently higher CKD prevalence among women in regions including China, Germany, Finland, Korea, Canada, the United States, and several European nations, despite notable regional variability. In contrast, countries such as Thailand and Japan have reported a lower prevalence of CKD among women compared to men [9,10]. Paradoxically, despite the higher prevalence of CKD in women, the incidence of end-stage renal disease (ESRD) is approximately 50% higher in adult men [11], a disparity that has been shown to widen over time and is largely driven by the rapid increase in diabetic nephropathy among older men, as demonstrated in long-term population-based studies conducted by Fukuhara et al. [12] in 5264 patients that initiated maintenance hemodialysis during the study period. Cause-specific analyses revealed that diabetic nephropathy was the primary driver of ESRD growth in both sexes, with a markedly steeper increase among men. Lastly, a USA-based cohort study of 3939 adults with CKD comparing men and women demonstrated that women had a lower risk of progressing to ESRD, experiencing a 50% decline in estimated glomerular filtration rate (eGFR) [13]. This discrepancy suggests sex-specific differences in disease progression, biological susceptibility, and healthcare utilization. Consistent with this trend, an increase in the number of men undergoing kidney replacement therapy (KRT), has continued to rise, potentially reflecting faster disease progression and differential biological responses in men [14]. These statistics highlight the urgent need for earlier detection and intervention, particularly in populations at higher risk of rapid disease progression.

Despite the widespread burden of CKD, timely detection of CKD stems from the limited availability of early predictive biomarkers that identify risk in asymptomatic individuals prior to substantial eGFR decline, particularly in primary care settings where routine blood testing is not always feasible, especially in remote areas. Currently, kidney function assessment relies on standard laboratory-based evaluations, including blood and urine tests that measure serum creatinine (SCr), cystatin C, albuminuria, and eGFR using established equations [15].

In contrast to many CKD studies published online that rely primarily on laboratory biomarkers and clinical measurements, increasing attention has been directed toward lifestyle and demographic factors as critical, yet often underutilized, determinants of CKD risk and progression. Lifestyle behaviors including dietary habits, physical activity, smoking, alcohol consumption, and sleeping pattern, along with demographic characteristics such as age, sex, and race, play a significant role in CKD development and long-term outcomes [16,17]. These factors are particularly important because they are largely modifiable and independently associated with kidney function, making them valuable targets for early risk stratification and prevention. For example, obesity and physical inactivity have been consistently linked to reduced kidney function and accelerated CKD progression [18], while smoking and excessive alcohol consumption further exacerbate kidney damage through inflammatory and hemodynamic mechanisms [19]. Additionally, underlying biological and social determinants, including age, sex, and race or ethnicity, also influence CKD susceptibility and progression rates [20], reinforcing the need for predictive approaches that extend beyond traditional clinical indicators. Our study innovates by demonstrating the relevance of these readily available lifestyle and demographic indicators.

### Machine Learning and CKD

Given the chronic and progressive nature of CKD, identifying high-risk individuals at an early stage before irreversible kidney damage occurs is essential to prevent or delay the need for dialysis or transplantation. Integrating lifestyle and demographic factors into predictive frameworks offers a promising avenue for early risk stratification. In particular, the use of machine learning (ML) techniques enables the analysis of complex, multidimensional data to uncover patterns that may not be apparent through traditional statistical approaches.

ML is recognized as transformative tool in public health that enables the analysis of large-scale, complex datasets to identify individuals at risk of CKD, hypertension, cancer and other conditions long before clinical symptoms appear, supporting early intervention and prevention. In nephrology, ML enhances CKD prediction and classification by integrating data types such as laboratory results, imaging, and patient medical history, which leads to more precise and accurate assessments of disease progression and prognosis [21,22]. Consequently, artificial intelligence (AI)-based early diagnostic and clinical decision-support systems have been increasingly developed and applied in healthcare settings [23].

Because early-stage CKD is typically asymptomatic, timely identification at the population level remains a public health priority. This study introduces a novel machine learning-based framework for the early detection of CKD in men by prioritizing lifestyle and demographic indicators rather than conventional and common laboratory-dependent biomarkers. Unlike much of the existing CKD prediction literature, which predominantly relies on biochemical parameters such as serum creatinine, eGFR, and albuminuria—often available only after kidney damage has progressed—this study focuses on readily obtainable, non-invasive, and modifiable lifestyle factors alongside key demographic characteristics. By doing so, the proposed approach shifts the emphasis from late-stage diagnosis to early risk identification within primary healthcare and community-based settings. Importantly, the proposed machine learning framework is designed for feasibility in low-resource healthcare settings, as it relies on routinely collected demographic, lifestyle, and basic laboratory variables commonly available in primary care. The selected models are computationally lightweight and can be implemented using standard desktop computers without specialized hardware or extensive technical expertise, making them suitable for deployment in economically constrained and under-resourced health systems.

Furthermore, this study addresses an important and underexplored population-level disparity by specifically targeting men, who, despite having a lower overall prevalence of CKD than women, experience a disproportionately higher incidence of end-stage renal disease.

## 2. Materials and Methods

### 2.1. Dataset

The dataset used in this study was constructed for CKD risk classification using open-source data from the National Health and Nutrition Examination Survey (NHANES) 2017–March 2020 pre-pandemic dataset, accessible at https://wwwn.cdc.gov/nchs/nhanes/ (accessed on 10 November 2024) Table 3. The CDC’s official combined pre-pandemic dataset (2017–2020) is fully processed, with survey weights for national estimates; while newer data (2021–2023) was released in October 2024, many key variables are incomplete. NHANES is a continuous program that collects cross-sectional data on the health and nutritional status of the U.S. population through interviews and physical examinations. It uniquely provides comprehensive lifestyle and sociodemographic information including detailed dietary intake, physical activity, sleep patterns, smoking, alcohol consumption, and routine laboratory measurements, making it particularly suitable for investigating early CKD risk through integrated lifestyle, demographic, and biochemical factors. In addition, NHANES provides heterogeneity, standardized measurement procedures, and reduced selection bias.

The raw data consisted of 11 domain-specific Comma-Separated Value (CSV) files covering demographic characteristics, anthropometrics, blood pressure, blood biochemistry, urinalysis, nutrient intake, physical activity, sleep, smoking, and alcohol consumption. All files were merged using a unique personal identifier (SEQN) as a common key. Each file was first checked for the presence of the SEQN variable, and the datasets were then integrated by an inner join to retain only complete cases without missing values across domains. A total of 33 CKD-related factors were collected, and additional variables such as estimated glomerular filtration rate (eGFR) and ACR were used for CKD staging. CKD labels were represented as 0 or 1, based on the calculated eGFR. This dataset enabled a comprehensive, multidimensional approach to predicting early-stage CKD using lifestyle, demographic, and biochemical factors (Table 3).

### 2.2. Preprocessing Method

In the preprocessing stage, data cleaning was conducted in two steps.

(i) First, we filtered the dataset to include only male participants aged between 30 and 60 years (RIAGENDR = 1, 30 < age ≤ 60), as this age group was the target of the analysis. Participants outside this age range were excluded.
(ii) To prevent unnecessary sample reduction in this relatively small dataset, missing values in categorical variables were encoded as an explicit *Unknown* category, enabling the model to learn patterns associated with missingness rather than excluding observations. Missing values in numerical variables were imputed using the median, a robust statistic less sensitive to outliers commonly present in clinical measurements.

Using this approach, a total of 968 samples were retained for analysis. To evaluate the generalization performance of the proposed model, 30% of the dataset was reserved as an independent test set, while the remaining 70% was used for model development. The training–validation subset was further subjected to 5-fold cross-validation, in which model training and validation were performed exclusively within this subset. After cross-validation, the final model with optimized parameters was applied to the independent test set to generate predictions and evaluate performance. The test set was not used at any stage of model training or hyperparameter optimization. Nevertheless, we acknowledge that the limited sample size may still predispose the models to overlearning, and therefore our findings should be interpreted cautiously and validated in larger independent cohorts.

For blood pressure, the mean of the first and second systolic/diastolic measurements (BPXOSY1/2, BPXODI1/2) was calculated to obtain representative values (BPXOSY, BPXODI). For dietary intake, the results of the first-day and second-day dietary recalls (DR1, DR2) were averaged to compute daily mean values of total calories (TKCAL), protein (TPROT), carbohydrate (TCARB), sugar (TSUGR), fiber (TFIBE), fat (TTFAT), sodium (TSODI), and potassium (TPOTA). Additional variables included body mass index (BMI), fasting glucose (LBXGLU), serum uric acid (LBXSUA), blood urea nitrogen (LBXSBU), serum creatinine (LBXSCR), urinary albumin (URXUMA), urinary creatinine (URXUCR), physical activity (PAQ620), sleep (SLD012), smoking (SMQ020), and alcohol consumption (ALQ111).

Among the merged data, only male participants aged >30 and ≤60 years were included in the analysis. This restriction was applied to control for sex- and age-related physiological differences and to focus on the high-risk CKD group of middle-aged male patients. The estimated glomerular filtration rate (*eGFR*) was calculated based on serum creatinine and age. The CKD outcome was defined using eGFR calculated from serum creatinine and age using the Chronic Kidney Disease Epidemiology Collaboration (CKD-EPI) equation:

(1) $$eGFR\;=\;141\times {\left(\frac{SCr}{0.9}\right)}^{\alpha }\;\times {\left(0.993\right)}^{Age}$$

where *SCr* is the serum creatinine concentration (mg/dL). When *SCr* ≤ 0.9, *a* = −0.411; when *SCr* > 0.9, *a* = −1.209. While these variables contribute to outcome construction, they were retained as input features to align model development with real-world clinical workflows in which serum creatinine and age are available at the time of assessment. For participants identified as non-Hispanic Black (RIDRETH1 = 4), a correction factor of 1.159 was applied to the estimates. The albumin-to-creatinine ratio (*ACR*) was computed as

(2) $$ACR=\frac{URXUMA}{URXUCR}$$

Based on eGFR, kidney function stages were defined at three levels: eGFR ≥ 90 → normal (G1); 60–89 → mildly decreased (G2); eGFR < 59 → moderately to severely decreased (G3a–G5) combined with the presence of kidney damage, as reflected by albuminuria or other clinical indicators of kidney dysfunction. Positive class: G2–G5 with kidney damage markers (ACR ≥ 30 mg/g or eGFR < 60); negative: G1 without markers. While G2 (eGFR 60–89) does not necessarily indicate CKD in the absence of kidney damage, G2 individuals who show signs of kidney damage are at increased risk of progression to more severe stages of CKD. Therefore, G2 was grouped with G3a–G5 as the CKD risk group (1) to ensure that individuals at higher risk, due to either reduced kidney function or the presence of damage, were included, while G1 was treated as the normal group (0), producing the final binary target variable CKD label. This approach aligns with standard clinical practice, where G2 with kidney damage is considered a CKD risk, and it reflects the need for earlier risk stratification and intervention. All missing or infinite values were removed, and the finalized dataset was saved as a .csv file.

The mean age of the participants was 46.2 years (standard deviation = 8.7). The mean body mass index (BMI) was 29.8 ± 6.8 kg/m^2^, indicating that most of the participants were overweight or obese. The average systolic and diastolic blood pressures were 124.8 ± 15.8 mmHg and 78.4 ± 11.1 mmHg, respectively. The mean fasting glucose level was 116.6 ± 45.3 mg/dL, with several participants showing hyperglycemic readings (Table 4).

In summary, the dataset used in this study represents a comprehensive CKD risk-prediction dataset integrating lifestyle, biochemical, nutritional, sleep, and activity factors among middle-aged men, all with complete and valid data records.

**Figure 1:**
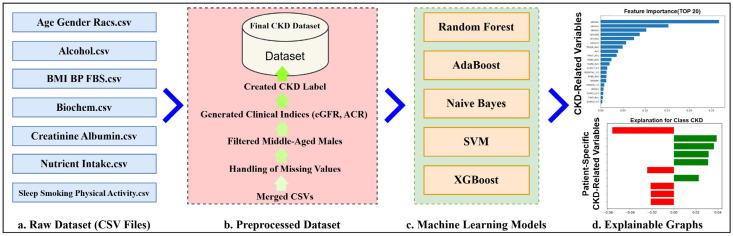
Overview of the data processing and model development pipeline.

As illustrated in Figure 1, multiple CSV files were loaded and subjected to the preprocessing steps described in process (b). Several CSV files were merged into a single dataset using the common identifier SEQN through an inner join operation. Missing values were subsequently addressed via feature-wise imputation or encoding, enabling model training without excluding observations. The dataset was then restricted to middle-aged male participants. Derived clinical indices, such as eGFR and ACR, were generated, and a final CKD label was constructed to form the complete dataset. In process (c), five representative machine learning models—Random Forest, AdaBoost, Naïve Bayes, SVM, and XGBoost—were applied and compared to evaluate their classification performance. Finally, as shown in process (d), explainable graphs, including Local Interpretable Model-agnostic Explanations (LIME), a Pearson heatmap, and SHapley Additive exPlanations (SHAP), were generated based on the best-performing model to provide additional clinical interpretability.

Ethical approval was not required for this study, as it utilized de-identified, publicly available data from NHANES, which has been approved by the National Center for Health Statistics Ethics Review Board

### 2.3. Instruments

In this study, five representative machine learning models, including Random Forest, AdaBoost, Naïve Bayes, Support Vector Machine (SVM), and XGBoost, were employed to compare their performance. These models were chosen because they are widely used in medical prediction studies, demonstrate robustness even in small-sample datasets, and enable comparison of the trade-off between predictive performance and model interpretability, which is critical for clinical and primary healthcare applications. All models were trained using the same set of lifestyle and demographic features derived from NHANES dataset to evaluate their ability to capture nonlinear relationships and interactions relevant to early CKD risk factors. This comparative framework ensures a fair assessment of model performance while aligning the analysis with the study’s aim of developing an interpretable and practical risk prediction approach suitable for primary healthcare settings.

#### 2.3.1. Random Forest

Random Forest, proposed by Breiman [24], is an ensemble learning algorithm that combines multiple decision trees to improve predictive performance. Each tree was trained independently using bootstrap samples of the data and a random subset of features, thereby introducing diversity among the trees. The final prediction is obtained by aggregating the outputs of all trees by averaging (for regression) or majority voting (for classification). This ensemble mechanism reduces overfitting and enhances the robustness against noise in the data. Recently, CKD studies have shown that Random Forest achieves high predictive value for assessment of risk factors associated with CKD, supporting its suitability for early prediction among individuals with risk factors. This has significant implications for early intervention and prevention in CKD [25].

#### 2.3.2. AdaBoost

Adaptive Boosting (AdaBoost), developed by Freund and Schapire [26] is a boosting algorithm that sequentially combines multiple weak learners to form a strong classifier. Initially, all samples are assigned equal weights; during training, the weights of the misclassified samples are increased so that the model focuses more on difficult cases. The final prediction is a weighted combination of all weak learners, allowing AdaBoost to achieve a high classification accuracy with relatively simple base models [26] and making it well suited for early CKD risk prediction tasks using lifestyle and demographic indicators. A study conducted by Ganie et al. [27] demonstrated AdaBoost to have the overall best performance in CKD prediction, scoring highest among performance measures, with 100% and 98.47% accuracy on the training and testing sets. It also exhibited optimum precision, recall, and area under the curve–receiving operator characteristic (AUC-ROC) curve performance.

#### 2.3.3. Naïve Bayes

The Naïve Bayes classifier is a probabilistic model based on Bayes’ theorem, which assumes conditional independence among features given the target variable. It estimates the posterior probability for each class and assigns a sample to the class with the highest probability. Despite its simplifying assumptions, Naïve Bayes is computationally efficient and performs robustly, even with high-dimensional or small-sample datasets such as the 968 NHANES cases analyzed in this study. It is particularly effective in clinical datasets where feature interdependencies are weak [28], as confirmed by its application in prior CKD prediction studies such as those by Priyanka et al. [29]. (94.6% accuracy) and Gudeti et al. [30], which highlighted its utility for early detection using heterogeneous risk factors like age, blood pressure, and albumin [31].

#### 2.3.4. Support Vector Machine (SVM)

The SVM, introduced by Vapnik, is a supervised learning algorithm that seeks an optimal hyperplane to separate data points of different classes. By maximizing the margin between classes, the SVM enhances the generalization performance. For nonlinear data, kernel functions, such as the radial basis function (RBF) or polynomial kernels, are employed to project the input data into higher-dimensional spaces, enabling complex pattern recognition. SVMs are particularly effective for high-dimensional, nonlinear, and noisy medical data, including CKD prediction tasks with heterogeneous features like those in the present NHANES analysis, where they have achieved accuracies up to 98.5% with feature selection [31,32,33].

#### 2.3.5. XGBoost

eXtreme Gradient Boosting (XGBoost), developed by Chen and Guestrin, is an optimized implementation of the gradient boosting framework designed for high performance and computational efficiency. It trains a sequence of decision trees, where each new tree corrects the errors of the previous ensemble by minimizing the differentiable loss function using gradient descent. The inclusion of regularization terms helps to prevent overfitting, whereas parallel learning and automatic handling of missing values improve scalability. Owing to these advantages, XGBoost has demonstrated outstanding accuracy and interpretability in various biomedical prediction tasks, including CKD detection, with up to 99.16% accuracy, eGFR decline forecasting, and renal injury prediction in gout patients using NHANES data [34,35,36]. A study conducted by Raihan et al. [35] on a CKD dataset from the University of California, Irvine (UCI) machine learning repository demonstrated the XGBoost classifier to obtain an accuracy of 99.16%, precision of 100%, recall of 98.68% and F1 score of 99.33% using 24 features.

### 2.4. Explainable Graphs

In this study, SHAP, LIME, Feature Ranking, and a Pearson correlation heatmap were applied to enhance the interpretability of the machine learning models. SHAP visualizes the contribution of each feature to the model’s output by providing a quantitative value for its impact on the final prediction. This method enables the transparent visualization of complex “black-box” models, such as ensemble algorithms [37]. LIME explains individual-level predictions (CKD = 0 or non-CKD = 1) by identifying which features influenced each specific case. It is particularly suitable for clinical decision support, allowing the analysis of how each factor affects CKD prediction in each patient [38,39]. Feature Ranking is an algorithm that evaluates the statistical importance of features, distinguishing meaningful predictors from those with negligible impact. This validates the significance of the variables and provides interpretability to the AI model results [40]. Finally, the Pearson correlation heatmap represents the linear relationships between the continuous variables. Visualizing the correlation coefficients in a heatmap helps assess the structural stability of the dataset and detect redundancy or multicollinearity among the variables.

## 3. Results

This study was conducted in a Linux-based server environment equipped with Python 3.10, CUDA 12.1, an RTX 4500 GPU, and 128 GB of RAM. The evaluation metrics included accuracy, precision, recall, and F1-score. In addition, interpretable graphs were analyzed to provide further insights.

### 3.1. Machine Learning Model Comparison

In this study, five machine learning models (XGBoost, Random Forest, SVM, Naïve Bayes, and AdaBoost) were implemented to predict the presence of chronic kidney disease (CKD). As summarized in Table 5, evaluation metrics including accuracy, precision, recall, and F1-score were compared across the models.

Among the evaluated models, AdaBoost demonstrated the best overall performance. AdaBoost achieved the highest accuracy (0.7285) among all models, while maintaining a well-balanced trade-off between precision (0.6050) and recall (0.6923), resulting in the highest F1-score (0.6457). These results indicate that AdaBoost can reliably identify individuals at risk of CKD while effectively limiting excessive false positive predictions.

The Random Forest model exhibited a relatively high recall (0.8173); however, its precision was limited to 0.5059, leading to a lower F1-score compared to AdaBoost. Similarly, the SVM model achieved the highest recall (0.8365) among all models but suffered from a substantially low precision (0.4046), indicating an imbalanced prediction performance. In clinical prediction settings, such behavior may lead to an increased false positive rate despite high sensitivity. In contrast, the Naïve Bayes model showed consistently lower performance across all evaluation metrics, suggesting limited applicability for CKD risk prediction.

**Figure 2:**
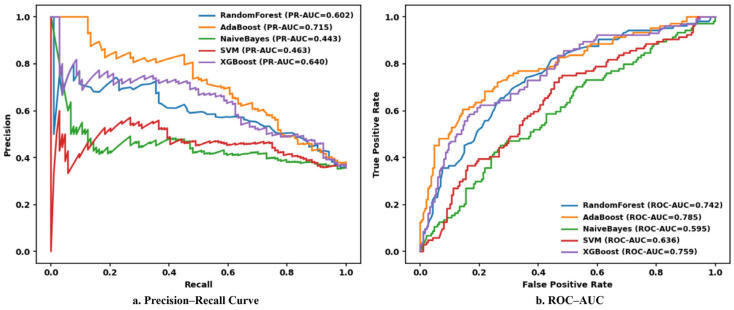
Comprehensive evaluation of model performance ((**a**) precision–recall curves among five classifiers; (**b**) ROC-AUC).

Figure 2a presents the precision–recall (PR) curves of the five machine learning models. AdaBoost achieved the highest area under the PR curve (PR-AUC = 0.715), followed by XGBoost (PR-AUC = 0.640) and Random Forest (PR-AUC = 0.602). In comparison, SVM (PR-AUC = 0.463) and Naïve Bayes (PR-AUC = 0.443) demonstrated substantially lower PR-AUC values, indicating reduced discriminative ability in the presence of class imbalance. These findings highlight the superiority of ensemble-based models, particularly AdaBoost, for identifying CKD risk cases.

Figure 2b illustrates the receiver operating characteristic (ROC) curves for all models. AdaBoost achieved the highest ROC-AUC value (0.785), followed by XGBoost (ROC-AUC = 0.759) and Random Forest (ROC-AUC = 0.742). In contrast, SVM (ROC-AUC = 0.636) and Naïve Bayes (ROC-AUC = 0.595) showed comparatively weaker classification performance.

Overall, AdaBoost consistently outperformed the other models across accuracy, F1-score, PR-AUC, and ROC-AUC, demonstrating robust and well-balanced predictive performance. Notably, AdaBoost showed superior generalization capability in a clinically relevant, class-imbalanced CKD dataset. Accordingly, AdaBoost was selected as the final model for subsequent explainability analyses using SHAP and LIME.

### 3.2. Explainable Graphs

**Figure 3:**
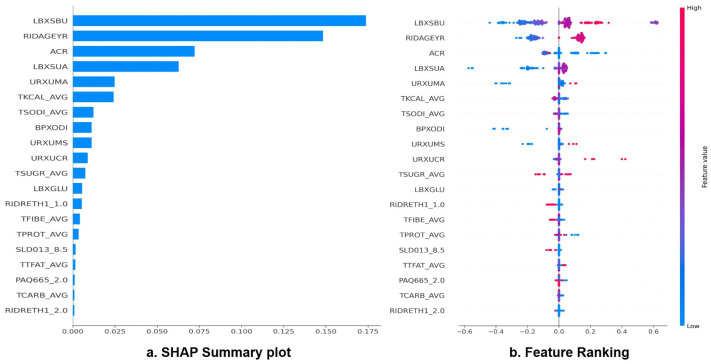
Explainability analysis of the AdaBoost model for CKD prediction.

The SHAP summary plot (Figure 3a) for the AdaBoost model revealed that blood urea nitrogen (LBXSBU) exhibited the highest mean absolute SHAP value, indicating that it was the most influential feature in predicting CKD, as supported in the study by Seki et al. [41], emphasizing that higher BUN levels were associated with adverse renal outcomes and may be a useful biomarker for predicting CKD progression. Blood urea nitrogen is a metabolic byproduct of protein catabolism that is excreted by the kidneys, and elevated levels reflect impaired renal excretory function. Clinically, BUN is widely used as a standard indicator for assessing kidney function, and its emergence as the top-ranked predictor in the model supports the strong clinical validity of the findings [42]. BUN elevations often precede creatinine rises in prerenal states and early tubular dysfunction, making it a sensitive marker for subclinical renal stress even when serum creatinine remains within normal limits. Examination of the SHAP value distribution further demonstrated that higher LBXSBU values (Figure 3b) consistently contributed positively to CKD predictions, indicating a direct association between elevated blood urea nitrogen levels and increased CKD risk.

The second most important predictor was age (RIDAGEYR) (Figure 3b). Although a gradual decline in eGFR with aging is often considered part of normal physiological senescence, Kidney Disease: Improving Global Outcomes (KDIGO) emphasizes that even age-related reductions in GFR are associated with higher cardiovascular and renal risk, which supports focusing on middle-aged men (30–60 years) as a clinically relevant target group for early risk stratification [2,43]. The SHAP distribution showed that advanced age contributed positively to CKD prediction, reflecting the well-established decline in glomerular filtration rate and reduced renal functional reserve associated with aging [44]. This finding is consistent with epidemiological evidence reporting a higher prevalence of CKD among older populations [45].

The albumin-to-creatinine ratio (ACR) emerged as the third most influential feature, indicating that ACR carries substantial weight in the algorithm’s discrimination between low- and high-risk individuals. Higher ACR values were consistently associated with positive SHAP contributions toward CKD classification, meaning that even modest elevations in ACR shifted individual predictions toward higher estimated CKD risk in our population. These findings align with evidence that even moderately increased albuminuria (KDIGO A2 category: ACR 30–300 mg/g or 3–30 mg/mmol) is an established marker of glomerular damage and is independently associated with adverse renal and cardiovascular outcomes, even in early-stage CKD [46,47]. By demonstrating that ACR is not only statistically important but also directionally consistent in its SHAP profile, our study reinforces the clinical relevance of incorporating ACR into ML-based screening strategies to flag individuals who may benefit from closer monitoring, risk factor modification, and earlier nephrology assessment.

In addition, serum uric acid (LBXSUA) was identified as an important contributor to the model. Elevated uric acid levels have been linked to renal dysfunction and CKD progression in previous studies, and uric acid’s prominence in the SHAP analysis aligns with existing clinical evidence regarding the role of hyperuricemia in kidney disease.

Several lifestyle and metabolic variables—including total caloric intake (TKCAL_AVG), dietary sodium intake (TSODI_AVG), urinary albumin (URXUMA), and diastolic blood pressure (BPXODI)—also contributed to CKD prediction, although to a lesser extent than primary biochemical markers. While their individual SHAP values were relatively smaller, these variables represent key modifiable risk factors with established clinical importance for CKD prevention and disease management. For instance, high dietary sodium intake promotes glomerular hypertension [48] and proteinuria progression. Overweight/obesity and sleep deprivation have been associated with an increased risk of CKD [49], urinary albumin signals early glomerular damage responsive to interventions, and elevated diastolic blood pressure reflects vascular stiffness amenable to lifestyle modification [50]. Meta-analyses further confirm that multicomponent lifestyle changes targeting diet, sodium restriction, and physical activity can reduce CKD incidence and slow progression, underscoring the value of these secondary predictors for personalized preventive strategies in primary care settings [51].

Furthermore, blood glucose (LBXGLU), dietary fiber intake (TFIBE_AVG), and protein intake (TPROT_AVG) demonstrated minor but non-negligible contributions to CKD prediction. Elevated fasting glucose and prediabetes are established risk factors for incident CKD and faster progression of existing disease [52]. Collectively, these findings support the notion that metabolic health and broader dietary patterns can influence renal function and CKD risk, even when their individual SHAP contributions are comparatively small.

Overall, the SHAP analysis indicates that the AdaBoost model primarily relies on established renal function and metabolic biomarkers such as blood urea nitrogen, age, and albumin-related indices while also incorporating the supplementary influence of lifestyle and dietary factors. This pattern suggests that the model captures clinically interpretable relationships rather than spurious statistical associations, supporting its potential utility in early CKD risk assessment and clinical decision-support systems.

**Figure 4:**
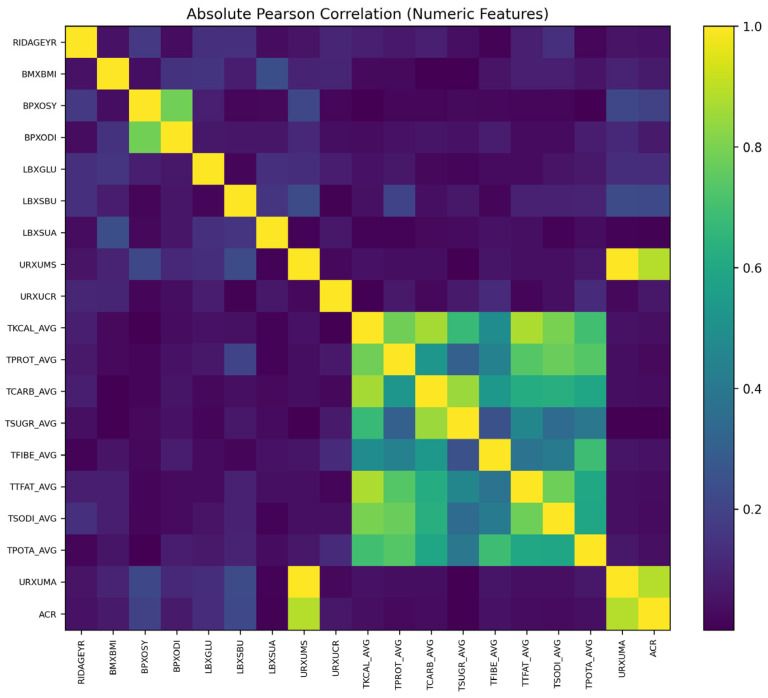
Absolute Pearson correlation heatmap of clinical and behavioral variables in the test dataset.

The absolute Pearson correlation heatmap (Figure 4) provides a quantitative overview of the linear relationships among the numerical variables used for CKD risk prediction. The color intensity represents the magnitude of the absolute correlation coefficient (|r|), with higher values indicating stronger linear associations between variables. Overall, most variable pairs exhibited correlation coefficients below 0.6, indicating the absence of strong multicollinearity across the dataset. This supports the stability of model training and enhances the reliability of model interpretation.

The most prominent correlation structure was observed among dietary intake variables. Total energy intake (TKCAL_AVG) showed moderate to strong correlations with carbohydrate (TCARB_AVG), protein (TPROT_AVG), fat (TTFAT_AVG), dietary fiber (TFIBE_AVG), sodium intake (TSODI_AVG), and potassium intake (TPOTA_AVG), forming a distinct cluster. This pattern suggests that overall dietary behaviors are closely interrelated, reflecting shared dietary habits rather than independent nutritional effects.

Limited correlations were also observed among renal function related biomarkers. In particular, urinary albumin (URXUMA) and albumin-to-creatinine ratio (ACR) exhibited a strong correlation, consistent with the fact that ACR is derived from urinary albumin excretion. In contrast, key biochemical markers such as blood urea nitrogen (LBXSBU), serum uric acid (LBXSUA), and blood glucose (LBXGLU) showed weak intercorrelations, indicating that each biomarker contributes distinct and complementary information for CKD risk assessment.

Demographic and clinical variables, including age (RIDAGEYR), body mass index (BMXBMI), and blood pressure (BPXOSY and BPXODI) [53], generally demonstrated low correlations with dietary and renal function variables (|r| < 0.4). This suggests that these variables contribute relatively independently to CKD risk prediction, and that the model does not rely excessively on any single group of features but instead integrates multiple heterogeneous risk factors.

In summary, the correlation analysis indicates that the dataset encompasses a well-balanced combination of dietary factors, renal biomarkers, metabolic indicators, and demographic characteristics, with minimal redundancy across feature domains. This structural property supports the robust learning behavior and high interpretability of the AdaBoost model, thereby strengthening the overall credibility and reliability of the study’s findings.

**Figure 5:**
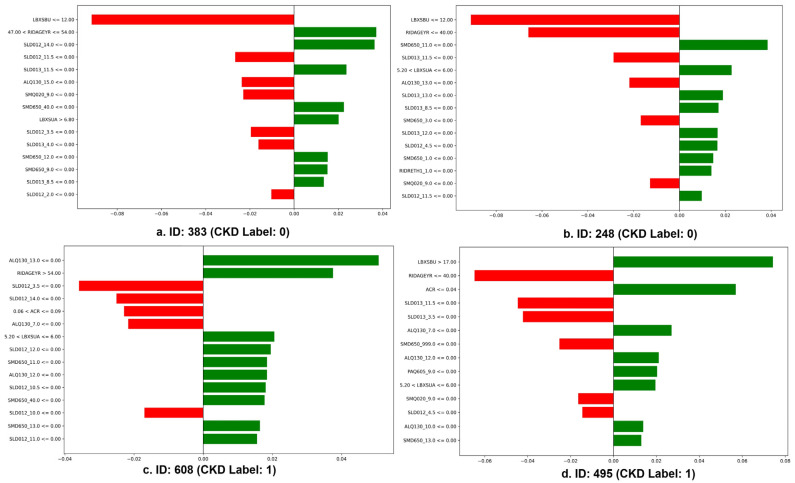
LIME-based local explanations for CKD prediction using the AdaBoost model (Green bars indicate increased CKD risk, whereas red bars indicate decreased CKD risk).

Figure 5 presents four representative LIME visualizations explaining individual-level predictions made by the AdaBoost classifier. Green bars indicate features that increase the probability of CKD (class = 1), whereas red bars denote features that decrease the predicted CKD risk. Importantly, the direction and magnitude of each contribution reflect the feature’s influence on the model output, regardless of the final predicted class.

(a,b) Non-CKD cases (ID: 383, ID: 248; CKD label = 0): In both non-CKD cases, low blood urea nitrogen (LBXSBU ≤ 12.0) was identified as the most influential factor reducing CKD prediction probability. Clinically, normal BUN levels indicate preserved renal filtration and excretory function, supporting a low risk of CKD. Younger age ranges (RIDAGEYR ≤ 40–54 years) also contributed negatively to CKD prediction, reflecting lower age-related renal vulnerability. Lifestyle-related variables, including sleep duration (SLD012, SLD013), physical activity (SMD650), and smoking status (SMQ020), showed heterogeneous contributions across cases, highlighting that even among non-CKD individuals, distinct behavioral profiles may differentially influence CKD risk estimation.

(c) CKD case (ID: 608; CKD label = 1): For the CKD-positive case, ID 608, advanced age (RIDAGEYR > 54 years) emerged as the strongest contributor increasing CKD prediction probability. This aligns with established clinical evidence linking aging with declining glomerular filtration rate and reduced renal reserve. Additionally, elevated uric acid (LBXSUA > 5.20) positively contributed to CKD risk, consistent with prior studies associating hyperuricemia with renal dysfunction and CKD progression. Sleep-related variables (SLD012, SLD013) and alcohol consumption (ALQ130) also showed positive contributions, indicating potential lifestyle-related risk amplification.

(d) CKD case (ID: 495; CKD label = 1): In ID 495, elevated blood urea nitrogen (LBXSBU > 17.0) was the dominant factor increasing CKD prediction probability, reflecting impaired renal clearance of nitrogenous waste products. Older age (RIDAGEYR > 40 years) and increased albumin-to-creatinine ratio (ACR > 0.04) further strengthened CKD prediction, suggesting the presence of glomerular damage and albuminuria. In contrast, certain lifestyle features exerted modest protective effects, illustrating heterogeneity in risk factor profiles even within CKD-positive individuals.

The results suggest that AdaBoost effectively differentiates normal kidney function from CKD risk patterns, leveraging core renal biomarkers, age, and supportive lifestyle metrics. LIME analysis demonstrates that the AdaBoost model primarily relies on clinically meaningful renal and metabolic biomarkers, particularly blood urea nitrogen, age, uric acid, and albumin-related indices, while also incorporating lifestyle-related features such as sleep, physical activity, and substance use. This combination enables individualized, interpretable predictions and supports the potential application of the model in early CKD screening and personalized risk stratification in real-world clinical and public health settings.

## 4. Discussion

This study demonstrates that lifestyle, sociodemographic, and biochemical variables enable robust prediction of CKD risk among middle-aged men (ages 30–60, *n* = 968) using nationally representative data. Among the machine learning models evaluated, AdaBoost demonstrated the highest performance (accuracy = 0.7258, F1-score = 0.6457, recall = 0.6923). The precision–recall curve (AP = 0.715) highlights the model’s reliability and strong generalization performance in distinguishing individuals at increased risk of CKD. While individual parameters like eGFR and albuminuria provide diagnostic thresholds, our ML approach integrates 33 heterogeneous factors to capture nonlinear interactions (e.g., combined effects of BMI, sodium intake, and sleep on creatinine levels) that are not captured by conventional rules. The AdaBoost model’s high recall and AP on independent test data demonstrate superior sensitivity for early risk detection compared to single-parameter assessment.

Interpretability was prioritized using multiple explainability techniques (SHAP, LIME, Boruta) and consistently revealed that classical renal function indicators such as serum creatinine, blood urea nitrogen, urinary creatinine, and age were the most influential CKD predictors. These features are clinically linked to glomerular filtration and kidney health, aligning with current nephrology guidelines and routine diagnostic algorithms. SHAP/LIME further provide patient-specific risk contributions, enabling actionable clinical decisions beyond traditional threshold-based interpretation.

Lifestyle and biological factors such as sleep duration, sodium and sugar intake, and BMI were also identified as secondary predictors, consistent with recent epidemiologic evidence. For example, SHAP and LIME analyses revealed patterns where high BMI combined with short sleep and elevated sodium intake shifted prediction contributions toward higher CKD risk, emphasizing actionable behaviors for modifiable risk factor management in primary care [54]. However, the absolute Pearson correlation heatmap showed weak to moderate relationships among most variables (|r| < 0.6) and clustered nutritional indicators (calories, carbohydrates, fat, protein, fiber), confirming a low-multicollinearity data structure, strengthening the transparency and stability of model inference.

The emergence of creatinine, urea nitrogen, age, urinary creatinine and related biomarkers as key predictors aligns with the CKD-EPI equation and established renal physiology. Notably, the finding that higher sodium and sugar intake and elevated BMI contribute to increased risk (from LIME/SHAP) is consistent with prior epidemiological and clinical evidence advocating for dietary and weight management strategies in CKD prevention. Furthermore, ensemble-based models (especially boosting models such as AdaBoost and XGBoost, and bagging models such as Random Forest) outperformed classical algorithms (SVM, Naïve Bayes), reaffirming previous reports that ensemble learning captures nonlinear and partially imbalanced structures in clinical data more effectively [55].

Key strengths of this study include: (1) focusing on a real high-risk group—middle-aged men; (2) integrating lifestyle, metabolic, behavioral, biochemical, and urinary markers in a unified preprocessing pipeline; (3) validating both performance and interpretability (global and local) to enhance clinical relevance and applicability; (4) practical feasibility of the proposed machine learning approach in low-resource healthcare settings; (5) practicality, as the algorithms employed are computationally efficient and can be integrated into existing electronic health record systems or deployed as standalone decision-support tools using open-source software. The high recall achieved supports sensitivity in at-risk patient, which is crucial for screening interventions. While this study demonstrates promising results for early CKD risk prediction in middle-aged men [56], several limitations warrant consideration. First, the model was developed using a male-only cohort aged 30–60 years from NHANES data, limiting direct generalizability among female or older adults, where CKD prevalence, progression rates, and risk factor profiles differ significantly. Second, the cross-sectional nature of NHANES data precludes causal inference, and longitudinal cohorts are needed to confirm predictive performance over time. Finally, while lifestyle factors emerged as secondary predictors, their assessment relied on self-reported measures subject to recall bias, underscoring the need for objective activity and dietary monitoring in future implementations. Future directions include sex-stratified models and validation across broader age groups, larger and more diverse cohorts being studies, and favorable feature-to-sample ratios, as well as employing principled multiple-imputation approaches to confirm robustness and generalizability.

In this study, we applied a rigorously controlled preprocessing strategy to address missing data and prevent information leakage between input variables and outcome labels, thereby minimizing potential bias during model training and evaluation. For categorical variables, an explicit *Unknown* category was introduced to preserve missing information without discarding samples, while numerical variables were imputed using median values to ensure stable learning without data loss. In addition, dependencies between variables used for CKD label generation and model inputs were carefully considered, allowing us to maintain clinical validity while avoiding leakage during the evaluation stage. This approach is particularly meaningful as it reflects the incomplete and heterogeneous nature of real-world clinical data while simultaneously enhancing model generalizability and reproducibility.

The tree-based ensemble models employed in this study, including Random Forest, XGBoost, and AdaBoost, are known for their robustness in the presence of correlated features and are well suited for clinical datasets that contain both raw and derived variables. Consistent SHAP and LIME analyses identified key renal function markers—such as blood urea nitrogen, serum creatinine, age, and urinary creatinine—as the most influential predictors of CKD. These findings align closely with established nephrology guidelines and renal physiology, indicating that the models learned clinically meaningful decision patterns rather than spurious correlations.

Beyond traditional renal biomarkers, this study also evaluated the contributions of modifiable lifestyle factors, including body mass index, sleep duration, and sodium and sugar intake, thereby extending CKD risk prediction from diagnostic assessment toward a preventive perspective. Local explanations derived from SHAP and LIME revealed that individuals sharing the same CKD label may exhibit distinct combinations and directions of contributing risk factors. This observation supports the feasibility of personalized risk profiling and targeted lifestyle interventions for CKD prevention.

Such an interpretable machine learning framework can be readily integrated into electronic health record (EHR) systems to support clinical decision-making, and its relatively low computational cost makes it suitable for deployment in resource-limited healthcare settings [57]. Future work will focus on validating the proposed approach in multi-center and multi-population cohorts to further assess generalizability. In addition, the framework may be extended to dynamic, time-series-based CKD risk prediction models, along with the development of patient-specific digital risk cards that visually summarize individual risk factors, thereby enhancing clinical usability and interpretability.

## 5. Conclusions

The integration of lifestyle, sociodemographic, and biochemical indicators from public health survey data enables accurate prediction of CKD risk among middle-aged men. Of the five machine learning models evaluated, AdaBoost yielded the most favorable predictive performance (accuracy = 0.7285, F1-score = 0.6457, recall = 0.6923), with robust precision–recall characteristics and consistent reliability across various classification thresholds. Explainability analyses employing SHAP, LIME, and Boruta consistently identified serum creatinine, blood urea nitrogen, urinary creatinine, and age as major contributors to CKD detection, aligned with clinical nephrology and routine renal laboratory tests. Lifestyle and metabolic indicators (i.e., BMI, sodium and sugar intake, and sleep duration) emerged as secondary factors for CKD but significantly support broader public health research on the role of modifiable behaviors in kidney disease development. These results indicate that ensemble-based models, particularly AdaBoost, can effectively capture the nonlinear and heterogeneous characteristics of clinical data. Furthermore, the interpretable outputs of the model (e.g., feature importance visualization) provided predictive insights consistent with established renal physiology, thereby strengthening clinical credibility and bridging the gap between predictive modeling and actionable patient care. This study deploys explainable artificial intelligence (AI) frameworks as decision-support tools among clinicians within electronic health record (EHR) systems. The high predictive accuracy of the models offers a transparent, feature driven explanation that can potentially enable personalized risk assessment and preventive intervention for CKD. The utilization of explainable AI for early CKD risk detection provides practical predictive frameworks for routine healthcare, paving the way for adoption in preventive and personalized medicine for at-risk populations in health management. Lastly, the low computational cost and reliance on routinely collected data support the scalability of this approach in resource-limited healthcare environments.

## 6. Recommendation

The proposed AdaBoost model is highly feasible for deployment across diverse healthcare settings, including low-resource primary care centers in economically challenged regions, due to its: (1) minimal laboratory requirements, (2) lightweight computational demands, (3) open-source deployment, (4) clinician-friendly outputs. Accordingly, future research in CKD risk prediction should focus on developing digital tools that are applicable in both private and public healthcare settings, integrating routine laboratory results with lifestyle, dietary, and sociodemographic information. These tools can enhance early detection, risk assessment, and personalized care in CKD [58]. Future studies should also explore the integration of additional clinical biomarkers, such as cystatin C, as well as genetic data and omics data, to improve the accuracy and predictive power of CKD risk models [59].

Meanwhile, by combining clinical biomarkers with self-reported behavioral factors (e.g., physical activity, sleep patterns, alcohol and tobacco use) and dietary intake, such tools may enable personalized and holistic CKD risk assessments [60]. These solutions could take the form of web-based calculators or mobile health (mHealth) applications integrated within electronic health record (EHR) systems. Integration with population health databases could further support risk stratification, health screening, and targeted interventions, while patient-facing applications may offer individualized prevention guidance. The development of clinical decision-making flowcharts for CKD risk classification and management may further assist clinicians in delivering personalized care, sup-porting earlier risk stratification, targeted prevention, and timely clinical intervention.

## Figures and Tables

**Table 1:** GFR categories in CKD.

GFR Category	GFR (mL/min per 1.73 m^2^)	Terms
G1	≥90	Normal or high
G2	60–89	Mildly decreased
G3a	45–59	Mildly to moderately decreased
G3b	30–44	Moderately to severely decreased
G4	15–29	Severely decreased
G5	<15	Kidney failure

**Table 2:** Albuminuria categories in CKD.

Category	AER (mg/24 h)	ACR (mg/mmol)	ACR (mg/g)	Terms
A1	<30	<3	<30	Normal to mildly increased
A2	30–300	3–30	30–300	Moderately increased
A3	>300	>30	>300	Severely increased

**Table 3:** Description of the variables used in the analysis.

Variable Name	NHANES Variables	Type	Class	Description
Age	RIDAGEYR	Numerical	Predictor	Age in years at screening
Gender	RIAGENDR	Categorical	Predictor	Patient’s sex (male, female)
Race	RIDRETH1	Categorical	Predictor	Race/Hispanic origin
Alcohol	ALQ111	Categorical	Predictor	Had at least 12 alcoholic drinks in any one year
	ALQ121	Categorical	Predictor	Had at least 12 alcoholic drinks in past 12 months
	ALQ130	Numerical	Predictor	Average number of alcoholic drinks per day—past 12 months
BMI	BMXBMI	Numerical	Predictor	Body mass index (kg/m^2^)
Blood Pressure	BPXOSY	Numerical	Predictor	Systolic blood pressure (mmHg)
	BPXODI	Numerical	Predictor	Diastolic blood pressure (mmHg)
Laboratory	LBXGLU	Numerical	Predictor	Fasting blood glucose (mg/dL)
	URXUMA	Numerical	Predictor	Urinary albumin (mg/L)
	LBXSBU	Numerical	Predictor	Blood urea nitrogen (mg/dL)
	LBXSCR	Numerical	Predictor	Serum creatinine (mg/dL)
	LBXSUA	Numerical	Predictor	Serum uric acid (mg/dL)
	URXUMS	Numerical	Predictor	Urinary methylmalonic acid (ng/mL)
	URXUCR	Numerical	Predictor	Urinary creatinine (mg/dL)
Dietary	TKCAL_AVG	Numerical	Predictor	Average total energy intake (kcal)
	TPROT_AVG	Numerical	Predictor	Average total protein intake (g)
	TCARB_AVG	Numerical	Predictor	Average total carbohydrate intake (g)
	TSUGR_AVG	Numerical	Predictor	Average total sugar intake (g)
	TFIBE_AVG	Numerical	Predictor	Average total dietary fiber intake (g)
	TTFAT_AVG	Numerical	Predictor	Average total fat intake (g)
	TSODI_AVG	Numerical	Predictor	Average sodium intake (mg)
	TPOTA_AVG	Numerical	Predictor	Average potassium intake (mg)
Physical Activity	PAQ605	Categorical	Predictor	Walked or bicycled for transportation (yes/no)
	PAQ620	Categorical	Predictor	Vigorous work activity (yes/no)
	PAQ650	Categorical	Predictor	Moderate work activity (yes/no)
	PAQ665	Categorical	Predictor	Walk or bicycle for work (yes/no)
Sleep	SLD012	Numerical	Predictor	Sleep duration on weekdays or workdays (hours)
	SLD013	Numerical	Predictor	Sleep duration on weekends or non-workdays (hours)
Smoking	SMQ020	Categorical	Predictor	Smoked at least 100 cigarettes in life (yes/no)
	SMQ040	Categorical	Predictor	Do you now smoke cigarettes? (yes/no)
	SMD650	Numerical	Predictor	How many days did you smoke in past 30 days?
	ACR	Numerical	Predictor	Calculated using Equation (2) (albumin–creatinine ratio (mg/g))
	GFR	Numerical	Predictor	Calculated using Equation (1); derived for labeling/predictors
	CKD	Binary	Target	G1 and G2 (1) or not/normal (0)

**Table 4:** Summary statistics of major variables in the CKD dataset.

Variable	Mean ± *SD*	Min	Median	Max	Note
Age (RIDAGEYR)	46.2 ± 8.7	31	47	60	Middle-aged males
BMI (BMXBMI)	29.8 ± 6.8	15.5	28.7	86.2	Overweight tendency
Systolic BP (BPXOSY)	124.8 ± 15.8	84	122	201	
Diastolic BP (BPXODI)	78.4 ± 11.1	47.5	77.5	143	
Fasting Glucose (LBXGLU)	116.6 ± 45.3	64	105	451	

**Table 5:** Comparison of ensemble and classical machine learning models on the test set.

Model	Acc	Precision	Recall	F1-Score
XGBoost	0.6391	0.4968	0.7500	0.5977
Random Forest	0.6494	0.5059	0.8173	0.6250
SVM	0.5017	0.4046	0.8365	0.5455
Naïve Bayes	0.5670	0.4191	0.5480	0.4570
AdaBoost	0.7285	0.6050	0.6923	0.6457

## Data Availability

Publicly available datasets were analyzed in this study. This data can be found here: https://wwwn.cdc.gov/nchs/nhanes/ (accessed on 10 November 2024).
